# Automated design of nighttime braces for adolescent idiopathic scoliosis with global shape optimization using a patient-specific finite element model

**DOI:** 10.1038/s41598-024-53586-z

**Published:** 2024-02-08

**Authors:** Aymeric Guy, Maxence Coulombe, Hubert Labelle, Soraya Barchi, Carl-Éric Aubin

**Affiliations:** 1 Polytechnique Montreal, 2500 Chemin de Polytechnique, Montreal, QC H3T 1J4 Canada; 2 Sainte-Justine University Hospital Center, Montreal, QC Canada; 3 Université de Montréal, Montreal, QC Canada

**Keywords:** Paediatric research, Paediatric research, Diseases, Biomedical engineering, Computer science

## Abstract

Adolescent idiopathic scoliosis is a complex three-dimensional deformity of the spine, the moderate forms of which require treatment with an orthopedic brace. Existing brace design approaches rely mainly on empirical manual processes, vary considerably depending on the training and expertise of the orthotist, and do not always guarantee biomechanical effectiveness. To address these issues, we propose a new automated design method for creating bespoke nighttime braces requiring virtually no user input in the process. From standard biplanar radiographs and a surface topography torso scan, a personalized finite element model of the patient is created to simulate bracing and the resulting spine growth over the treatment period. Then, the topography of an automatically generated brace is modified and simulated over hundreds of iterations by a clinically driven optimization algorithm aiming to improve brace immediate and long-term effectiveness while respecting safety thresholds. This method was clinically tested on 17 patients prospectively recruited. The optimized braces showed a highly effective immediate correction of the thoracic and lumbar curves (70% and 90% respectively), with no modifications needed to fit the braces onto the patients. In addition, the simulated lumbar lordosis and thoracic apical rotation were improved by 5° ± 3° and 2° ± 3° respectively. Our approach distinguishes from traditional brace design as it relies solely on biomechanically validated models of the patient’s digital twin and a design strategy that is entirely abstracted from empirical knowledge. It provides clinicians with an efficient way to create effective braces without relying on lengthy manual processes and variable orthotist expertise to ensure a proper correction of scoliosis.

## Introduction

Adolescent idiopathic scoliosis (AIS) is a complex three-dimensional (3D) deformation of the spine that affects 2 to 4% of the pediatric population and onsets after 10 years of age^1^. AIS tends to progress during the peripubertal growth spurt, as the magnitude of the scoliotic curves increases and the deformity worsens. This pathomechanism of progression can be biomechanically explained by the Hueter–Volkmann principle describing how bone growth is favored in relative tension on the convex side of the deformity and inhibited in relative compression on its concave side, leading to vertebral wedging over time^2^. This is commonly referred to as the scoliosis vicious cycle^3^. AIS patients progressing over 45° of Cobb angle generally require an instrumentation and spinal fusion surgery, an invasive procedure with undesirable side-effects such as a loss of mobility and potential complications over time^4^.

Bracing is the most common conservative treatment aiming at controlling this progression to avoid a surgical intervention. Braces are generally prescribed for full-time wear between 18 and 23 h/day for an average treatment duration of 2 years, or until skeletal maturity. Most take the form of thoracolumbosacral orthoses: rigid plastic shells that apply corrective forces onto the patient’s torso at predetermined contact points using tensioned closure straps^5^. It is hypothesized that this correction may contribute to break away from the scoliosis vicious cycle by aligning the spine in-brace and symmetrizing the compressive forces acting on the vertebral epiphyseal growth plates^3^. Bracing has been documented as an effective treatment when compared to observation alone^6^.

However, brace-wear compliance is far from optimal, even if it is strongly linked to treatment success. Most centers report compliance rates on the order of 60%^7,8^, with an average daily brace wear of around 13 h across patients^6,9^. To mitigate this poor adherence to treatment, nighttime braces can be prescribed and are generally better accepted by patients because they have a lesser impact on daily activities. Nighttime braces are only worn during sleep and incorporate more aggressive correction features to compensate for the decreased wear time. As a result, pronounced trunk rotation and shoulder imbalance can lead to comfort issues^10^, while effective correction of thoracic curves is limited due to the intrinsic stiffness of the ribcage^11^. Studies have shown comparable effectiveness between full-time and nighttime braces^12,13^, but more high-quality trials are required to confirm their equivalence^14^. Nevertheless, nighttime braces are a useful compromise to ease patients into the brace treatment.

Nowadays, braces are designed using a computer-assisted design/manufacturing approach (CAD/CAM)^15^, where a torso surface topography scan is imported in a CAD software in which an orthotist models contact and relief regions to create a deformed positive mold of the patient’s trunk^5^. This mold is carved by a numerical router in a block of foam around which a plastic sheet is thermoformed. The plastic shell is then trimmed, straps are added, and the brace is finally fitted on the patient in the clinic to ensure comfort. While manufacturing is generally similar across centers, various brace design approaches and corrective strategies exist^16^. Significant variability is observed between these sometimes-conflicting biomechanical concepts, and brace quality depends on many factors including the orthotist’s expertise, the chosen design protocol, and the overall treatment strategy^17^. Current braces are therefore mainly designed using empirical knowledge acquired over many years of experience, difficult to transfer outside of a single clinical center. In addition, published clinical trials contain several biases and heterogeneous patient cohorts that render a structured comparison of each approach impossible^18^. To this day, the best brace design method is still unknown^16^, and standardization remains insufficient to ensure a repeatable, quality treatment across centers with variable expertise^17,19^.

Many studies have correlated different alignment metrics to treatment success, but few provide in-depth biomechanical assessments of the effects of brace wear. Greater in-brace correction in the coronal plane—Cobb angles of the main thoracic (MT) and thoracolumbar/lumbar (TL/L) curves, greater axial vertebral rotation (AVR) correction in the transverse plane around the apex of the curve, and a focus towards preserving or increasing the healthy sagittal curves—thoracic kyphosis (TK) and lumbar lordosis (LL), were shown to be predictive of treatment success^20–22^. As a result, orthotists need to balance correction in the three anatomical planes to ensure an effective clinical impact. They usually aim at achieving a minimum of 50% correction of the main curve’s Cobb angle^14^, while simultaneously trying to move the spine into a normal sagittal alignment, generally to avoid brace-induced flatback, and reduce some of the deformity’s rotational component^19^.

A few research groups have created numerical tools to study and better understand brace biomechanics in 3D^23,24^. Specifically, our group has extensively developed a patient-specific finite element model (FEM) constructed from the patient’s torso surface topography scan, the brace’s 3D model, and a 3D reconstruction of the patient’s spine, ribcage, pelvis and sternum based on biplanar standing radiographs^25–27^. This FEM, previously validated using clinical data^26^, can simulate brace donning and tightening and was employed to study the impact of brace design features in a structured approach with minimal biases^17,28^. It was subsequently adapted to simulate nighttime bracing^29,30^. In parallel, an analogous FEM was also developed to simulate vertebral growth modulation of progressive deformities in AIS^31^, which was further adapted for fusionless surgical applications^32,33^, and validated for bracing specifically[34].

Our bracing FEM has been implemented in a clinical setting to assist orthotists in brace design^9,35, 36^. In a randomized controlled trial, 120 patients were separated into two groups: one with standard CAD/CAM braces, and one with CAD/CAM braces that had been further improved using the patient-specific FEM. In the latter, orthotists would design a brace, simulate it on the digital patient, modify their design based on the simulated correction, cutaneous pressures, and skin-to-brace distances, and repeat the process until a satisfactory design was achieved. The braces created were lighter, thinner, and covered less surface on the torso, but were not found to be different from standard CAD/CAM braces, in terms of correction of the scoliotic deformity, brace-wear compliance, or quality of life^9^. Adding manual improvements steps using the FEM lengthened the quantity of work per brace required from the orthotist, roughly 30 min per design iteration, which limited the process to an average of three iterations in practice^35^. Also, the design modifications were chosen and implemented by the orthotist, such that the final braces still converged towards their usual empirical corrective strategies with minor improvements. This trial confirmed the clinical utility of using a patient-specific FEM for brace design, but the full capacity to optimize correction while providing an effective useable tool transposable to many centers remains to be established.

To address these challenges, the objective of this work was to develop a new automated design method producing nighttime braces independently from empirical knowledge, and validate its clinical efficacy on a patient cohort. Our hypothesis was that a global shape optimization process leveraging a patient-specific FEM could autonomously create braces with sufficient in-brace correction (over 50%) without the need for any manual design input.

## Methods

### Cases and initial data for clinical validation

Seventeen skeletally immature AIS patients between 10 and 16 years of age and prescribed with a nighttime brace for an expected treatment duration of 2 years were prospectively recruited between December 2021 and August 2022. Inclusion followed the Scoliosis Research Society standardized criteria: no prior treatment, skeletal maturity Risser score between 0 and 2, main curve Cobb angle measured at the presenting visit between 20° and 45°^37^. The study protocol was approved by our Institutional Review Board (Sainte-Justine University Hospital Center, Montreal, Canada), all experiments were performed in accordance with the Declaration of Helsinki, and all patients and their parents and/or legal guardians provided written informed consent.

At each patient’s initial visit, a standing 3D surface topography of the torso was acquired using an infrared structured light sensor (Structure Sensor, Occipital Inc. Boulder, CO, USA), as well as standing biplanar radiographs using a calibrated low-dose digital radiography system (EOS System, EOS Imaging, Paris, France). From these radiographs, a 3D reconstruction of the spine, ribcage, pelvis and sternum was constructed using a previously described parametric registration method based on transversal and longitudinal inferences^38^.

For each patient, a brace was designed using a global shape optimization method leveraging a patient-specific FEM. Except for two manual alignment steps requiring rapid user input (< 5 min), the totality of design steps were automatically executed by an algorithm managing every step of the process in the Matlab environment (Matlab R2021a, MathWorks, Natick, MA, USA), and its execution time was measured. Depending on the flow of patient recruitment, this execution was parallelized on a single personal computer, up to four simultaneous patients.

### Creation of the patient-specific FEM

For each patient, the torso’s 3D surface scan was superimposed on the skeleton’s 3D reconstruction in two steps, first by automatically aligning the axes of the principal components of variance calculated for each point cloud, then by manually fine-tuning the alignment to ensure concordance with the clinical images. They were then used to generate a previously described and validated patient-specific FEM^26,34, 39^, briefly summarized here.

**Figure 1:**
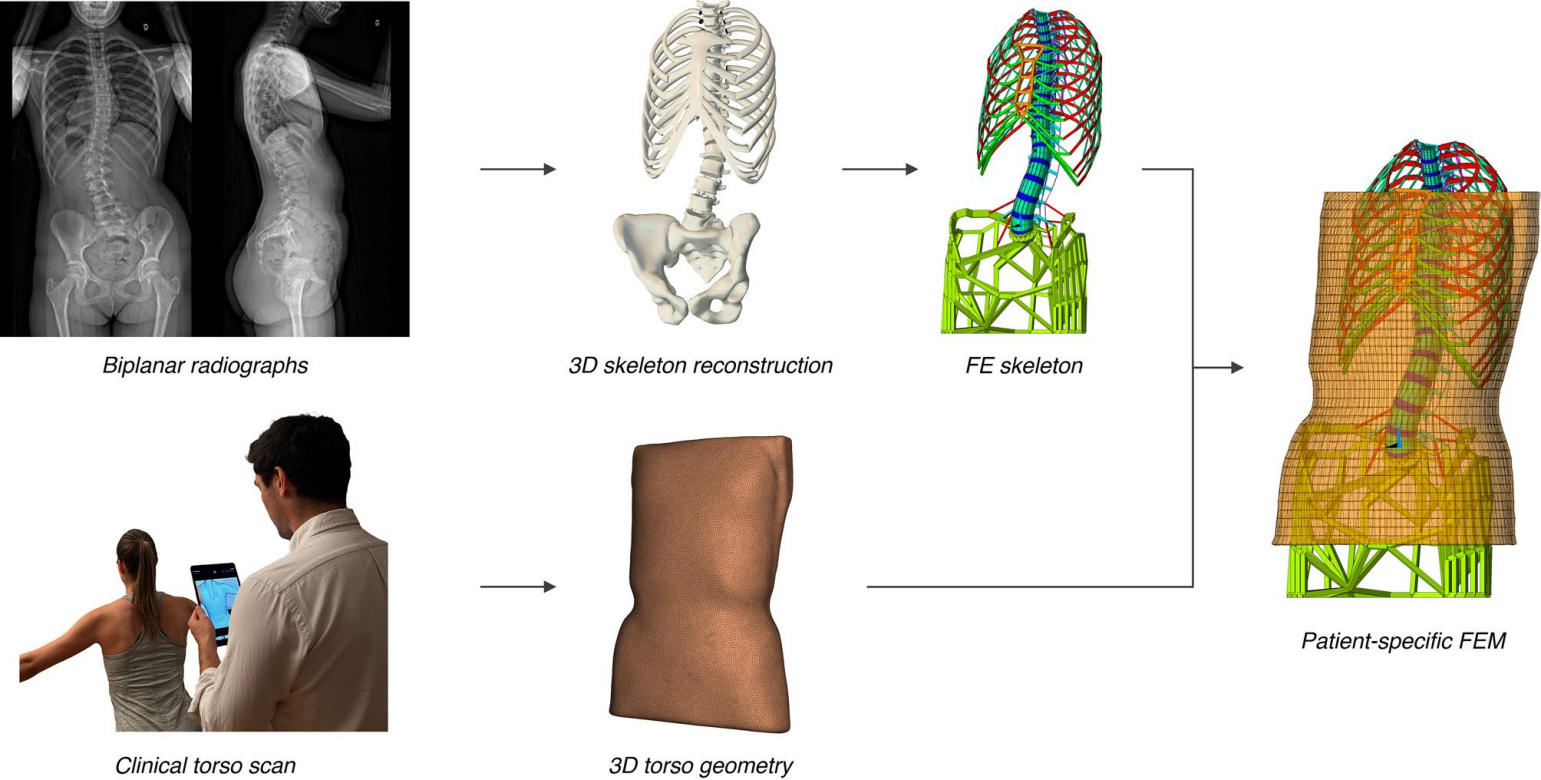
Creation of the patient-specific FEM from standard biplanar radiographs and a surface topography scan of the torso. Elements representing the internal soft tissues and connecting the torso skin to the skeleton are not shown for clarity.

The resulting registered patient geometry was imported in a finite element analysis software (ANSYS Mechanical 2020 R1, Ansys Inc., Canonsburg, PA, USA). A global coordinate system was defined such that the x-axis pointed anteriorly, the y-axis pointed left laterally, and the z-axis pointed cranially. Osseous structures were modeled as hexahedral structural solid elements (thoracic and lumbar vertebral bodies, intervertebral disks) and elastic beam elements (vertebral processes, ribs, sternum, and pelvis). Ligaments, internal soft tissues and joints were modeled as tension springs and beam elements. The torso skin geometry was represented by shell elements with constant thickness. Material properties of all anatomical structures were taken from experimental cadaveric studies^40,41^. An estimate of spinal flexibility based on the manipulation of the patient’s torso by the treating orthopedist at clinical evaluation was factored into the intervertebral disk stiffness. The FEM creation is illustrated on Fig. 1.

### Automatic generation of braces

#### Initial brace definition

Using the patient-specific FEM, vertebra T1 was first aligned with the centroid of L5’s endplate to correct coronal and sagittal imbalances, its x and y displacements were fixed to only permit craniocaudal movement, and all degrees of freedom of the pelvis were blocked. Displacements (${u}_{i}$, where $i=$ x, y and z directions) were then imposed onto the nodes corresponding to the left ($L$) and right ($R$) pedicles of vertebrae T2 to L4 with the goal of mirroring their coordinates with respect to the sagittal plane to achieve maximal over-correction (Eq. 1). A weight $W$ factored the amount of applied displacement to the desired value: $W$ = 0 corresponded to the initial undeformed geometry, $W$ = 0.5 to a spine perfectly aligned onto the sagittal plane, and $W$ = 1 to a fully overcorrected spine with an inverted deformity.

1 $$\begin{array}{c}{Applied \;displacements= W*\left( \begin{array}{c}{u}_{{x}_{L}}=\left({x}_{R}-{x}_{L}\right)\\ {u}_{{x}_{R}}=\left({x}_{L}-{x}_{R}\right)\\ {u}_{{y}_{L}}=-\left({y}_{R}+{y}_{L}\right)\\ {u}_{{y}_{R}}=-\left({y}_{L}+{y}_{R}\right)\\ {u}_{{z}_{L}}=\left({z}_{R}-{z}_{L}\right)\\ {u}_{{z}_{R}}=\left({z}_{L}-{z}_{R}\right)\end{array} \right)}_{T2-L4 pedicles}\end{array}$$

**Figure 2:**
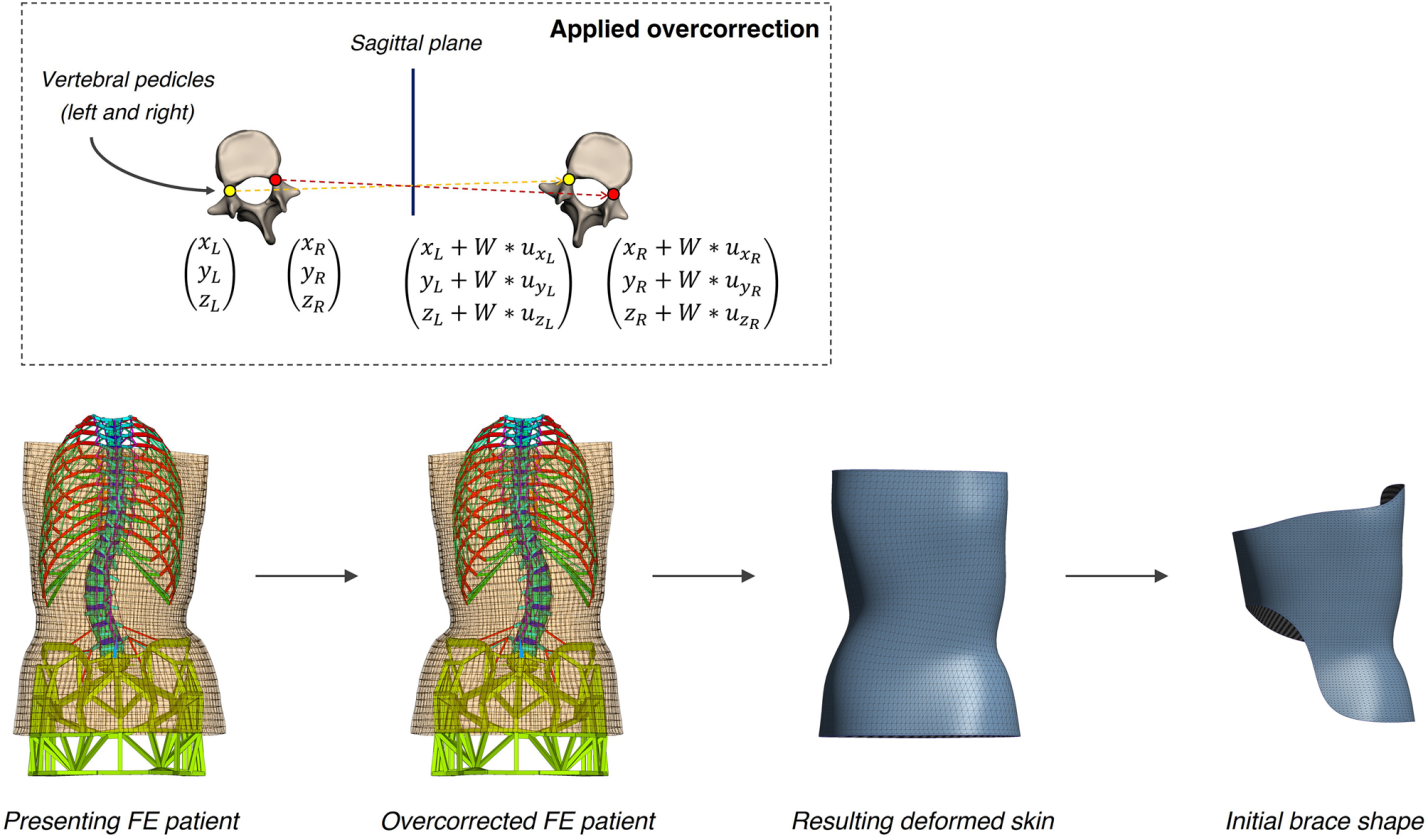
Generation of the initial brace shape: top row represents a top view closeup of a single vertebra and illustrates the applied overcorrection, achieved by imposing displacements according to Eq. 1 onto the vertebral pedicle nodes (L for left, R for right). Over-correction weight W is increased iteratively from 0 to 1, or until element distortion exceeds the FEM threshold. Bottom row represents a posterior view of the FE patient onto which a maximal over-correction (Wmax) is applied. The skin deforms in response to the applied displacements and the resulting geometry is cut by fitted splines to create the inner surface of the initial brace shape. Translucency was added to the skin elements (bottom left & center-left) to view the internal structures. Beam and spring elements representing internal soft tissues and connecting the torso skin to the skeleton were not shown for clarity.

In a stepwise manner, weight $W$ was increased from 0 to 1 by increments of 0.1. At every step, the finite element model was solved, and displacements of the vertebral pedicles caused the connected internal structures and the skin to deform. $W$’s increase was stopped if any element distortion in the deformed geometry exceeded the default program-controlled thresholds for convergence. The deformed skin corresponding to the maximal achieved overcorrection was extracted and converted to a stereolithography (STL) format. Finally, superior and inferior limits were added by manually aligning a spline onto the STL to create the cuts: superior limits were drawn to cover the trunk up to the axilla on the convex side of the thoracic deformity, and inferior limits to cover the trochanter on the ipsilateral side while leaving the contralateral iliac crest free. The resulting geometry was used as the internal surface of the initial brace shape in the optimization process (Fig. 2).

#### Optimization-modified brace shapes

**Figure 3:**
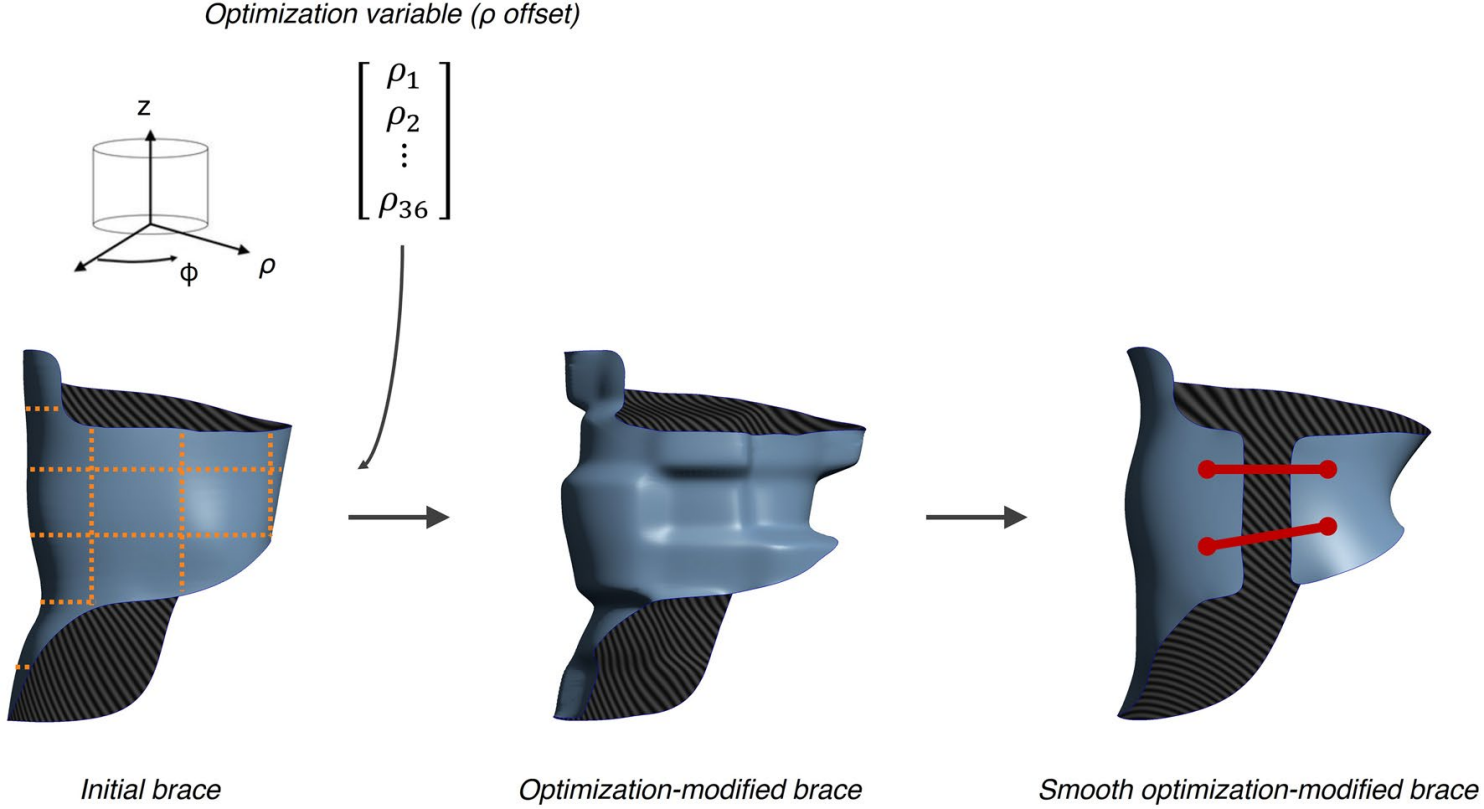
Modification of the brace shape by the optimization process (anterior view). A 6 × 6 grid (orange dotted lines) in cylindrical coordinates separated the brace surface into patches and optimization variable ρ offset translated their coordinates in the radial direction. The resulting brace was then smoothed, a frontal opening was created, and straps were added automatically.

The optimization process modified the brace topography to generate new shapes at every iteration (Fig. 3). To do so, the initial brace geometry was first imported as a point cloud. Coordinates were converted from the cartesian to the cylindrical coordinate system and the brace points were separated into patches (subsections) according to a cylindrical 6 × 6 grid in the z and φ directions. The radial (ρ) coordinates of the points in each patch were offset by modifiable values: a negative ρ offset translated the ρ coordinates of all patch points closer to the origin (pressure area) and a positive ρ offset translated these points further from the origin (relief area). This ρ offset vector, of length equal to the total number of patches (36), was the variable vector controlled by the optimization process. To constrain the optimization inside safe limits, the range of possible ρ offsets was set at [− 25, + 25] mm.

Once the ρ offset vector was applied to the brace points, the geometry was smoothed to fuse the offset slices together and remove aggressive topographical asperities, a frontal opening was created by removing the points falling into an arc of 20° in the φ direction centered around the frontal axis, and two or three straps were placed by automatically selecting points on each side of the opening falling closest to a proportional normalized height vector of [0.33, 0.66] or [0.25, 0.5, 0.75].

### Evaluation of brace biomechanical effectiveness

For each generated brace, the patient-specific FEM simulated the standing out-of-brace position, the supine position with the nighttime brace, and the subsequent growth modulation for the expected treatment duration of 2 years.

#### Standing out-of-brace simulation including gravitational loads (OOB)

**Figure 4:**
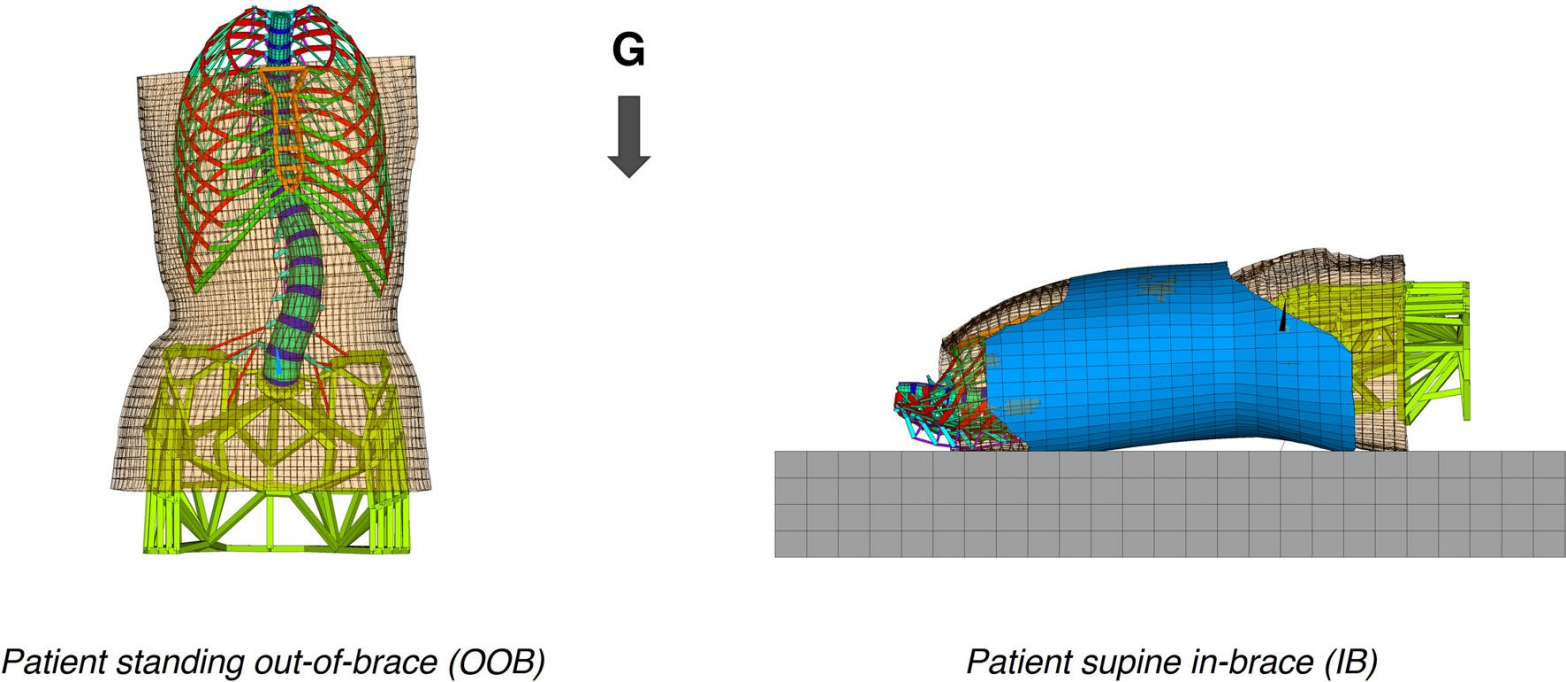
FE simulation of the two patient configurations: on the left, the standing out-of-brace position under gravitational (G) loads (OOB, anterior view); on the right, the supine in-brace position (IB, lateral right view). Translucency was added to the skin elements to view the internal structures. Beam and spring elements representing internal soft tissues and connecting the torso skin to the skeleton were not shown for clarity.

The FEM initially created modeled the patient’s standing geometry with no stresses acting on the anatomical structures. To determine the standing out-of-brace geometry under gravitational loads, as well as the patient’s weightless geometry and the stabilizing muscular forces in the standing position, a previously described and validated method was employed and is briefly summarized here^42^. Upwards gravitational forces were applied to simulate weightlessness with additional stabilizing muscular forces acting antero-posteriorly and laterally on vertebrae T6, T10 and L3. The resulting stresses were annulled, and downwards gravitational forces were then imposed. The stabilizing muscular forces were automatically tuned so that the loaded standing spine geometry conformed to the patient’s actual reconstructed spine. After solving, the standing loaded out-of-brace (OOB) patient geometry was obtained (Fig. 4).

#### Nighttime in-brace simulation in the supine position (IB)

To simulate nighttime bracing, the model previously described by Sattout et al. was refined^30^. The weightless patient geometry was imported and defined in the supine position. A mattress was modeled underneath using a grid of hexahedral solid elements with material properties of polyurethane foam (E = 0.3 MPa, ν = 0.2)^43,44^. The previously generated 3D model of the brace was added, modeled by hexahedral solid elements with the material properties of high-density polyethylene (E = 1 GPa, ν = 0.4) and a constant thickness of 4mm. Pairs of surface contact elements following the augmented Lagrangian formulation were created between the mattress and the skin, the internal surface of the brace and the skin, and the external surface of the brace and the top surface of the mattress. Gravitational forces were added, equivalent to the weight of the patient’s trunk evenly distributed across vertebral gravitational centers. The lateral and craniocaudal displacements of T1 and the pelvis were blocked, and the straps were tightened at 60N. The model was solved using a nonlinear static solver with linearly interpolated loads using the unsymmetric Newton–Raphson method. After solving, the supine in-brace (IB) patient geometry was obtained (Fig. 4).

#### Growth simulation

Stresses acting on each node composing the superior and inferior vertebral body epiphyseal growth plate of T2 to L5 were extracted from the two simulated configurations (OOB and IB). To obtain the nodal stresses averaged over the treatment period ($\sigma$), the OOB stresses in the standing position (${\sigma }_{OOB}$) and the IB stresses in the supine position (${\sigma }_{IB}$) were combined in the following formula (Eq. 2), including a compliance factor ($C$) of 0.33 indicative of the proportion of time spent in-brace during sleep (8 h per day):

2 $$\begin{array}{c}\sigma ={\sigma }_{OOB}+C*{( \sigma }_{IB}- {\sigma }_{OOB}) \end{array}$$

To quantify the asymmetrical stress distribution, these stresses were averaged on the left (${\sigma }_{L}$) and right (${\sigma }_{R}$) sides of each growth plate. Calculations of the local growth rates in response to stresses were performed following the formula^45^:

3 $$\begin{array}{c}{\left(\begin{array}{c}{G}_{L}={G}_{m}\left(1+\beta \left({\sigma }_{L}-{\sigma }_{m}\right)\right)\\ {G}_{R}={G}_{m}\left(1+\beta \left({\sigma }_{R}-{\sigma }_{m}\right)\right)\end{array}\right)}_{T2-L5\, growth\, plates} \end{array}$$

where ${G}_{L}$ is the longitudinal local growth rate applied on the left side of the growth plate, ${G}_{R}$ on the right side, ${G}_{m}$ is the baseline vertebral growth rate (0.8 mm/year for thoracic vertebrae, 1.1 mm/year for lumbar vertebrae)^31^, $\beta$ is the documented vertebral bone stress sensitivity factor (1.5 MPa^-1^)^45^, and ${\sigma }_{m}$ is the average stress on the entire growth plate.

**Figure 5:**
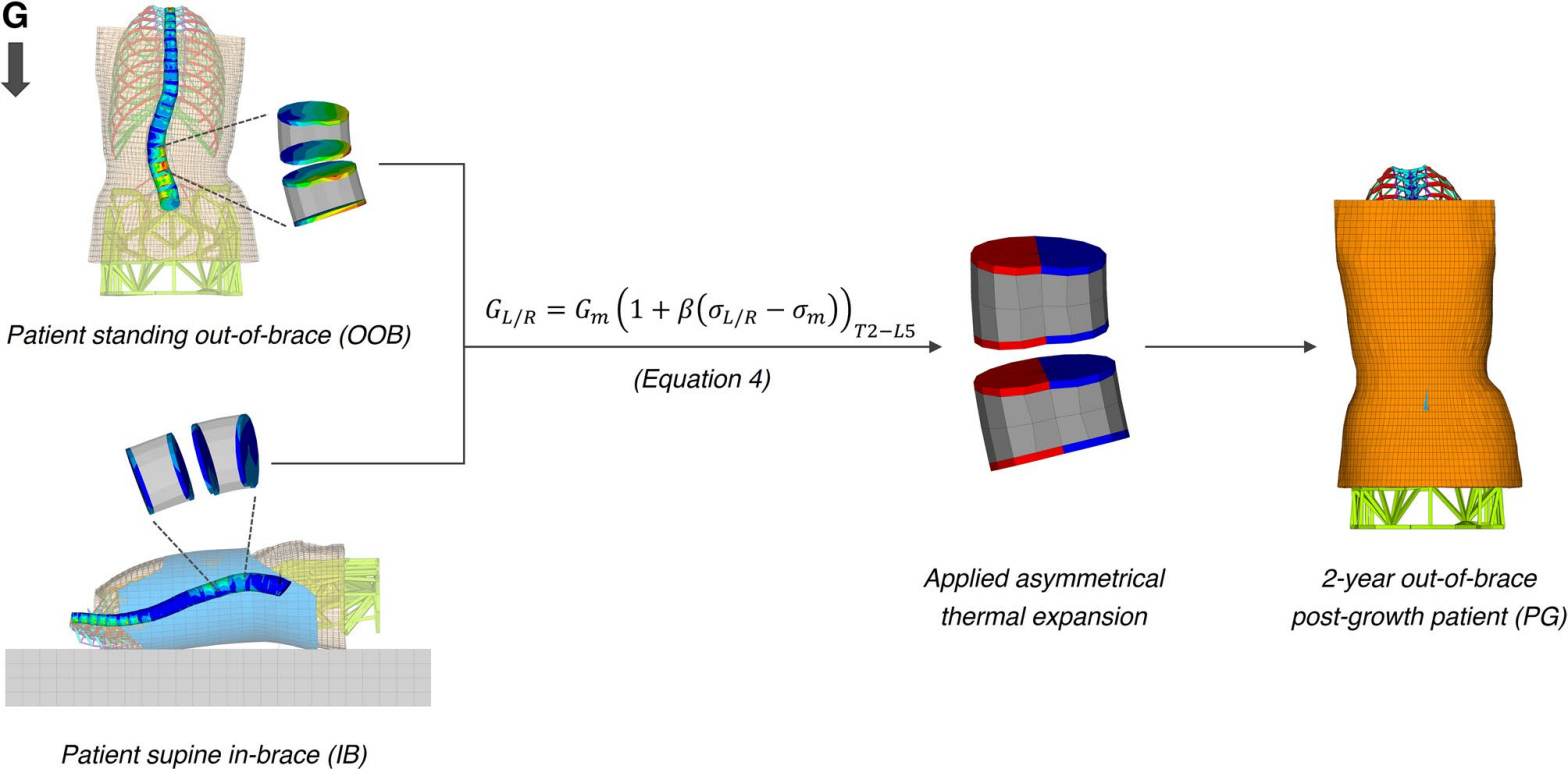
FE simulation of growth: the OOB (top left) and IB (bottom left) nodal stresses acting on the vertebral epiphyseal growth plates are combined to determine the amount of thermal expansion (center) applied on the left (red) and right (blue) nodes of each vertebral epiphyseal growth plate according to the growth rate formula (Eq. 3). After solving, the 2-year out-of-brace post-growth patient geometry is obtained (right). Closeups of the vertebral bodies of L2 and L3 are shown as examples. Other internal structures and posterior vertebral processes were not shown for clarity.

From the initial unloaded standing out-of-brace patient geometry, an asymmetrical thermal expansion corresponding to the calculated growth rate ${G}_{L}$ and ${G}_{R}$, multiplied by 2 years of treatment, was imposed on the left and right nodes of each growth plate respectively. After solving, the out-of-brace 2-year post-growth (PG) patient geometry was obtained (Fig. 5).

The FEM, including its immediate and post-growth simulations, was recently validated on a cohort of 35 patients following the rigorous ASME V&V40:2018 standard framework^34^. The difference between the simulated 3D deformity metrics and the real clinical measurements were < 6°, on the order of the measurement interoperator reproducibility.

#### Objective function

The supine in-brace (IB) and out-of-brace 2-year post-growth (PG) spine geometries were used to calculate the optimization’s objective function. To ensure an effective correction of the spine in the coronal and transverse planes while preventing flattening of the sagittal curves and favoring a normal alignment, metrics related to each anatomical plane of deformation were included in the objective function:

4 $$\begin{array}{c}OF={W}_{IB}*{\phi }_{IB}+ {W}_{PG}*{\phi }_{PG} \end{array}$$

where $OF$ is the objective function score, ${W}_{IB}$ and ${W}_{PG}$ are the weights factoring the simulated in-brace and post-growth configurations (5 and 10). The deformity terms ${\phi }_{IB}$ and ${\phi }_{PG}$ are calculated using the weighted sum:

5 $$\begin{aligned} \phi_{sim} & = W_{c} *\left( {\left| {\frac{{Cobb _{MT, sim} }}{{Cobb _{MT,ini} }}} \right| + \left| {\frac{{Cobb _{TLL,sim} }}{{Cobb _{TLL,ini} }}} \right|} \right) \\ & \quad + W_{s} *\left( {\left| {\frac{{\left| {TK_{sim} } \right| - |TK_{n} |}}{{\left| {TK_{ini} } \right| - \left| {TK_{n} } \right|}}} \right| + \left| {\frac{{\left| {LL_{sim} } \right| - \left| {LL_{n} } \right|}}{{\left| {LL_{ini} } \right| - \left| {LL_{n} } \right|}}} \right|} \right) \\ & \quad + \begin{array}{*{20}c} {W_{t} *\left( {\left| {\frac{{ AVR_{ MT,sim} }}{{AVR_{ MT,ini} }}} \right| + \left| {\frac{{AVR_{ TLL,sim} }}{{AVR_{ TLL,ini} }}} \right|} \right) } \\ \end{array} \\ \end{aligned}$$

where $sim$ is the simulated configuration (IB or PG), Cobb is the Cobb angle, $ini$ is the initial value measured on the presenting deformity, $MT$ is the main thoracic curve, $TLL$ is the thoracolumbar/lumbar curve, $TK$ is the thoracic kyphosis, $LL$ is the lumbar lordosis, $AVR$ is the average of the axial rotation of the curve’s three most rotated vertebrae (apex ± 1 level), and ${W}_{c}$, ${W}_{s}$, ${W}_{t}$, are the weights factoring the coronal, sagittal and transverse alignment metrics respectively (2, 1 and 1). To penalize only the misaligned sagittal curves, the following values are affected to ${TK}_{N}$ and $L{L}_{N}$^46^, based on their documented normal range^47^:

$${TK}_{n}= \left\{\begin{array}{c}20^\circ \\ {TK}_{sim}\\ 40^\circ \end{array} \begin{array}{c}if\; {TK}_{sim}< 20^\circ \\ if \;20^\circ <{TK}_{sim}<40^\circ \\ if {TK}_{sim}> 40^\circ \end{array}\right.$$

6 $$\begin{array}{c}{LL}_{n}= \left\{\begin{array}{c}30^\circ \\ {LL}_{sim}\\ 60^\circ \end{array} \begin{array}{c}if \;{LL}_{sim}< 30^\circ \\ if \;30^\circ <{LL}_{sim}<60^\circ \\ if \;{LL}_{sim}> 60^\circ \end{array}\right. \end{array}$$

### Optimization process

As formulated, diminishing OF scores were linked to better biomechanical effectiveness according to generalized clinical objectives. Therefore, the optimization’s goal was to find the global minimum of this OF score by modifying the ρ offset vector controlling the brace topography. In addition, safety constraints related to the contact pressure between the brace and the skin were added to prevent braces designed too aggressively (maximum localized nodal contact pressure < 450 kPa, the documented threshold of barely perceptible pain in the MT & TL/L regions^48^). Iterations where the maximal in-brace contact pressure exceeded the set threshold were deemed invalid.

A surrogate optimization algorithm was used to carry out the optimization process^49^. This algorithm involves two main phases: constructing a surrogate and searching for a minimum. Initially, it generates random points within defined bounds and evaluates the objective function at these points to create a surrogate using a radial basis function interpolator. Then, it searches for the function's minimum by sampling numerous points, evaluating a merit function using the surrogate, and choosing the best candidate for evaluation by the objective function. This adaptive point updates the surrogate for further iterations. The algorithm balances between minimizing the surrogate and exploring new areas by adjusting the search scale and using different sampling methods.

Parameters including minimum surrogate points, grid size for brace patches and convergence criteria were tuned via experiments on test patient data, with the objective of striking a balance between search broadness, consistency and total solve time (< 7 days): the surrogate was constructed with a minimum of 72 random points using the initial brace topography as seed, and it was stopped after 500 iterations if convergence was reached (< 5% OF score improvement over more than 50 iterations). If not, its execution was extended until convergence was attained, up to 1000 iterations.

### Brace manufacturing and clinical evaluation

**Figure 6:**
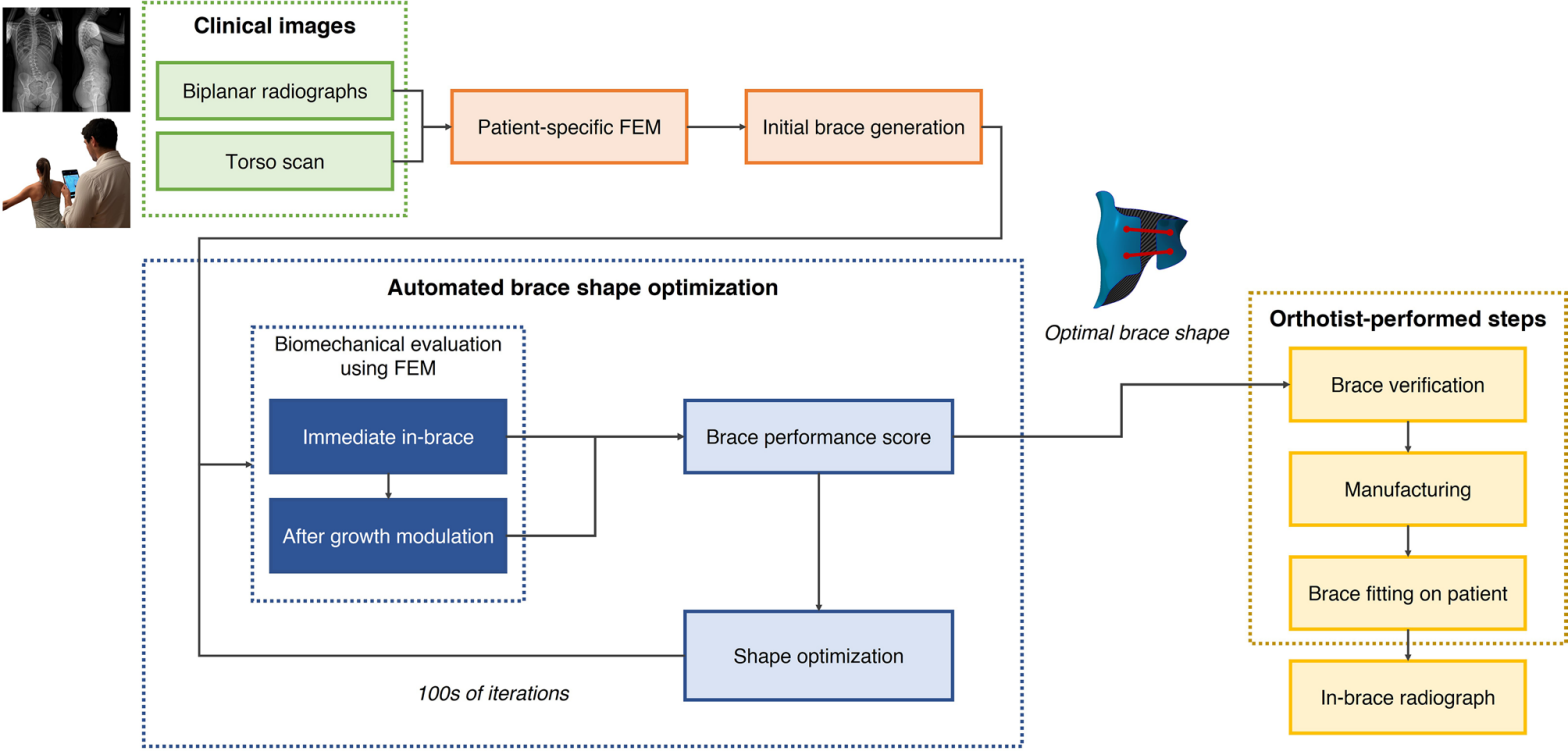
Complete design workflow repeated for all patients. From the standard clinical images, the patient-specific FEM is created and used to evaluate the automatically generated brace shapes. The design modifications are guided by the optimization process aiming to minimize the OF score. Each brace generated by this method was verified, manufactured and fitted on the patient. An antero-posterior in-brace supine radiograph was acquired on the same day.

Figure 6 summarizes the entire design and manufacturing workflow. All optimal braces created by the algorithm were first verified and approved by the treating orthotist, then manufactured using their usual CAM approach: the uncut topography of the optimized brace was sent to a numerical milling machine to create its positive mold, and a 4mm thick high-density polyethylene sheet was thermoformed around it. Velcro straps were affixed at the locations set by the algorithm.

The resulting braces were finally fitted onto each patient at a clinical visit 3 weeks after the initial one. If needed, minor flaring and sanding of the brace’s edges was performed by the orthotist to ensure comfort, but these modifications did not affect the brace’s topography. On the same day, an anteroposterior supine in-brace radiograph was acquired to measure the in-brace Cobb angle correction. To validate the design approach, the simulated correction was analyzed in 3D at each major step of the method and compared in the coronal plane to the actual in-brace correction. Finally, the predicted 3D correction after 2 years of simulated growth was calculated and expected clinical outcomes were derived.

### Statistical analyses

Comparison of 3D metrics was achieved using paired *t* tests, with a 0.05 significance level. Normality was verified using Shapiro–Wilk tests, and homogeneity of variances for each pairwise comparison was verified using F-tests. If normality was not demonstrated, non-parametric Wilcoxon signed-rank tests were used instead. All statistical analyses were performed in the Matlab environment (Matlab R2021a, MathWorks, Natick, MA, USA).

## Results

**Figure 7:**
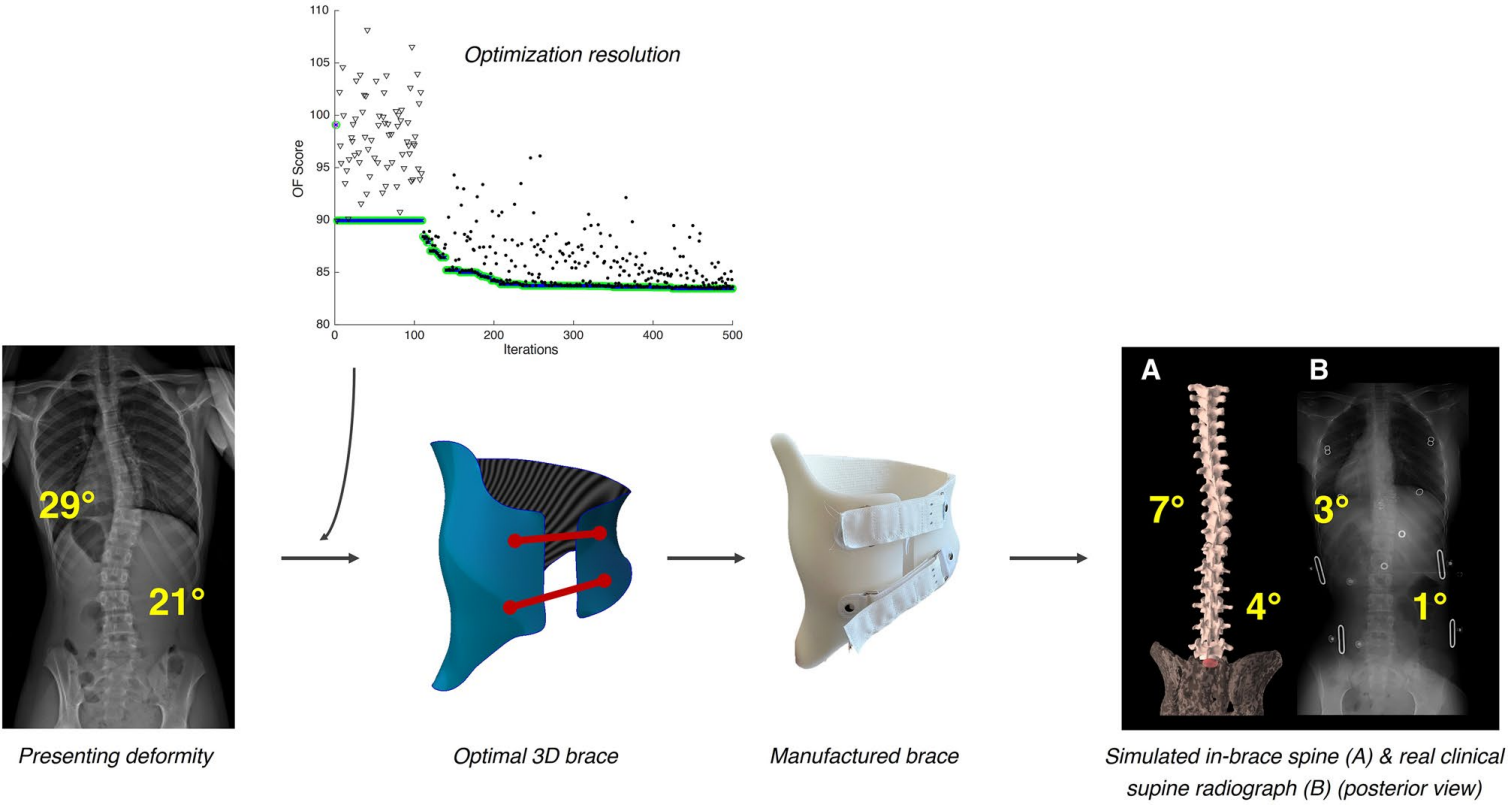
Optimization graph (top center) showing the evolution of OF scores across the 500 optimization iterations following the surrogate optimization algorithm for a typical patient (bottom left). The resulting optimal brace (bottom center) was manufactured and fitted on the patient. The simulated spine geometry was compared to the actual clinical radiograph (bottom right).

All recruited patients received an optimized brace, approved with no modifications by the orthotist. All braces were well fitted and comfortable according to verbal testimonies of the orthotist and the patients. The average hidden time for the algorithm to execute on a single personal computer was 136 ± 28 h, or 5.7 ± 1.2 days. Figure 7 shows an example of a typical patient’s optimization results and the simulated in-brace correction compared to the actual one measured on the supine radiograph.

**Figure 8:**
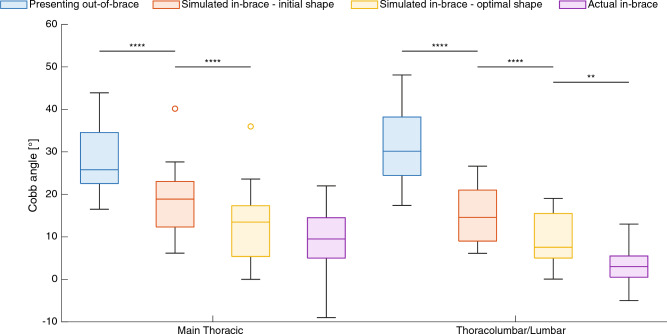
Cobb angle evolution for all patients: presenting out-of-brace deformity (blue), pre-optimization initial brace simulation (orange), post-optimization optimal brace simulation (yellow) and actual in-brace correction measured on the supine radiographs (purple). Negative values imply an over-correction. Statistically significant differences from paired *t* tests are indicated with **(*p* < 0.01) or ****(*p* < 0.0001).

Table 1 presents the measured 3D deformity metrics at the presenting visit, measured or simulated values inside the optimal brace, and simulated after 2 years. For all 17 patients, the initial presenting out-of-brace Cobb angles were 28° ± 8° (MT) and 31° ± 9° (TL/L) (Fig. 8). Donning the initial brace (pre-optimization) reduced the simulated in-brace Cobb angles to 19° ± 9° (MT) and 15° ± 6° (TL/L) (*p* < 0.0001). Donning the optimal brace (post-optimization) improved the correction to 13° ± 9° (MT) and 9° ± 6° (TL/L) (*p* < 0.0001). The actual Cobb angles measured on the patient’s in-brace radiographs with the manufactured optimal brace were 9° ± 8° (MT) and 4° ± 5° (TL/L), corresponding to an actual in-brace correction of 70 ± 28% (MT) and 90 ± 15% (TL/L), or a main curve correction of 82 ± 23%.

**Table 1:** Initial, in-brace and 2-year deformity metrics, measured and simulated. Results are presented as mean ± standard deviation [range]; values based on clinical radiographs measurements are indicated with a (M), values simulated using the FEM are indicated with a (S).

	Initial	Immediate in-brace	2-year post growth
Cobb MT (°)	28 ± 8 [17,44] (M)	9 ± 8 [0, 22] (M)	27 ± 15 [10,56] (S)
Cobb TL/L (°)	31 ± 9 [17,48] (M)	4 ± 5 [0, 13] (M)	32 ± 10 [16,52] (S)
Thoracic kyphosis (°)	27 ± 11 [13,44] (M)	16 ± 8 [3,33] (S)	28 ± 10 [13,48] (S)
Lumbar lordosis (°)	42 ± 19 [16, 77] (M)	37 ± 15 [17, 67] (S)	44 ± 23 [15, 91] (S)
Axial rotation MT (°)	5 ± 4 [1,16] (M)	3 ± 3 [0, 10] (S)	6 ± 5 [0, 19] (S)
Axial rotation TL/L (°)	8 ± 7 [0, 22] (M)	8 ± 7 [0, 21] (S)	8 ± 7 [0, 23] (S)

The supine position flattened the sagittal curves at baseline, and adding the optimal brace in that position did not significantly modify the simulated TK; however, it improved the LL by + 5° ± 3° (*p* < 0.0001). In the transverse plane the simulated axial rotation of the MT apical vertebra was corrected by 2° ± 3° (*p* = 0.036) but stayed unchanged for the TL/L apical vertebra (*p* > 0.05).

The post-growth out-of-brace simulations showed a non-significant average Cobb angle evolution of − 1° ± 8° (MT) and + 1° ± 5° (TL/L). In 12 patients (71%), the deformity would improve or stabilize (± 5°), while in the remainder cases it changed by less than 16% (MT) and 33% (TL/L).

## Discussion

This work describes a novel automated design method leveraging a patient’s digital representation, able to create effective personalized braces from standard clinical images with no prior empirical biases. To our knowledge, it is the first time a patient-specific finite element model was used to this extent for bespoke orthosis design in a real clinical setting. Other groups have developed computer-assisted workflows for similar devices but have not incorporated the iterative evaluation of their biomechanical effects to guide design decisions^50,51^.

Braces created using our method were biomechanically effective. In-brace correction of the main scoliotic curve systematically exceeded the 50% correction threshold. Recent studies on nighttime bracing report average in-brace corrections ranging from 61 to 85%^52,53^, which puts our measured in-brace corrections in the upper portion of the range, or even higher. While an acceptable correction was achieved solely via the computationally inexpensive pre-optimization brace shape, the optimization process significantly improved the correction further and appears to be an essential step in the method. In 3D, transverse plane correction was limited compared to the coronal plane, as generally observed in other studies^54,55^, and sagittal curves were preserved inside a normal range as the brace-induced flatback effect was adequately avoided or even slightly improved for the lumbar lordosis. In a meta-analysis analyzing outcomes at the end of treatment, Buyuk et al. pooled nighttime bracing success rates (Cobb angle progression < 5°) at 59%, lower than our simulated prediction of 71%^13^.

Immediate simulated corrections with the optimal brace were inside the clinically accepted threshold of the actual clinical radiographs (< 5° average difference), but our FEM systematically underestimated the in-brace correction, especially in the TL/L region. This may be due to flexibility factors that were overestimated and need to be more finely tuned to the patient’s individual characteristics. Despite the differences, the immediate simulation predictions were sensitive to brace topographical variations and the prediction offsets did not significantly affect the differential values used to guide the optimization process. Our model was therefore deemed valid for a comparative context of use like the one employed in this method.

In addition to immediate correction, the incorporated growth simulation provides information on how a specific brace influences the mechanisms of deformity correction over time. This represents an additional tool that can be used not only by the optimization process to account for long-term effects, but also by the clinical team to better prepare patients for treatment according to its expected evolution. In an analogous study, Cobetto et al. have shown that a growth model can be a powerful tool in assisting clinical decision-making and planning, leading to effective long-term results^33^.

While our immediate in-brace corrections were almost twice greater than the ones seen in full-time braces^56^, the reduced amount of prescribed daily wear limits the achievable effectiveness of the brace over longer treatments, as highlighted by our 2-year simulated outcomes. This prediction, however, does not include other rehabilitation activities that can be juxtaposed with the treatment’s effect to increase longer-term efficacy. Nevertheless, brace-induced growth modulation is only active a third of the time, in a period where gravity has less impact on the deformity due to the supine position during sleep. Many questions remain on the performance of nighttime bracing only, and our initial post-growth predictions support the conclusion that higher levels of brace wear (more than 8 h/day), for instance by adding a component of daytime bracing, may need to be combined to our design workflow to achieve significant improvements in long-term outcomes^6^. In this sense, our method is easily adaptable to full-time braces, as only the in-brace simulation step needs to be simplified to shift from the supine to the standing position.

Our method removes brace design from traditional empirical protocols. These approaches tend to categorize patients based on their presenting characteristics, as suggested by the growing number of scoliosis classifications published and employed^16,18, 57^. However, every patient differs, and the optimal treatment strategy can rarely be implemented by following hard rules within rigid categories. In the field of artificial intelligence, recent developments in reinforcement learning have shown that algorithms can be more performant if they do not rely on data biased by human experience, but rather on well-crafted reward functions^58^. In a similar fashion, our process has no root in empirical design knowledge to influence where and how corrective features are placed. It is fully tailored to each patient’s unique deformity and relies solely on their biomechanical simulations and a clinically driven objective function to achieve an optimal design. In our study, this function and its constraints were generalized according to documented factors linked to treatment success^21,22^. Future work should investigate modifying its weights to target the deformity components more specifically on a case-by-case basis, to further personalize the desired correction.

Our study presents a few limitations. First, to limit the exposure of children to excessive radiation, only a coronal supine radiograph was taken inside the manufactured brace, on the same day as brace delivery. This prevented an accurate 3D reconstruction of the spine in-brace, which did not allow the real sagittal and transverse corrections to be measured. Also, patients were not yet tracked over time, rendering the evaluation of post-growth corrections and long-term outcomes impossible. Further validation of the 3D correction and the long-term effects will be analyzed in a subsequent prospective trial, where patients will be followed until skeletal maturity, with objective compliance monitoring and quality of life assessments.

To reduce the number of confounding variables in our evaluation, manufacturing was kept traditional, using the established CAM method of thermoforming. However, the digital nature of our approach is strongly suited to additive manufacturing processes, which could be further automated. 3D printing is becoming widely used in orthoses other than scoliotic braces, and a few implementations of printed braces are currently being validated with promising results^59,60^. Future work should combine our proposed method with additive manufacturing to truly automate the brace creation process.

Overall, our method changes the way orthotists currently work. The traditional CAD/CAM process often requires hours of cognitively intensive manual or computer-assisted shape design, even more when combined with additional steps to obtain performance feedback before fabrication^35^. Our workflow shifts this demanding work from the orthotist to a machine that can run constantly without interruption. By simultaneously executing up to four patients on a single personal computer, we’ve shown that it is also parallelizable. Greater computing power and additional improvements to increase resolution speed and parallelization would reduce the machine-time needed to obtain an optimized brace using our method and provide an interesting potential for scalability. Orthotists could therefore allow more of their time on design validation, comfort adjustments, and patient management. As expert orthotists specialized in brace design for scoliosis grow rare, this could lead to increased productivity and efficiency in clinical logistics, with significant cost reductions at scale.

Finally, effective and standardized brace design protocols with optimal treatment management are difficult to implement in multiple centers with diverse practices. As a result, they can lead to variable execution and outcomes depending on each specific clinical set-up^19^. As few leading centers fine-tune their approach based on experiential improvements, others with less expertise, resources or in remote areas cannot benefit from these developments. Our personalized automated design approach, fully tailored to any patient’s individual characteristics and implementable remotely, aims at addressing these limitations while increasing access to reliable and effective treatments worldwide.

## Data Availability

Measurements and analysis done at the Sainte-Justine University Hospital Center are on a password protected server. Access may be arranged through application to the Research Ethics Board. Data access requests should be addressed to Carl-Éric Aubin.
